# Management of antineutrophil cytoplasmic antibody–associated vasculitis with glomerulonephritis as proposed by the ACR 2021, EULAR 2022 and KDIGO 2021 guidelines/recommendations

**DOI:** 10.1093/ndt/gfad090

**Published:** 2023-05-10

**Authors:** Marta Casal Moura, Philipp Gauckler, Hans-Joachim Anders, Annette Bruchfeld, Gema M Fernandez-Juarez, Jürgen Floege, Eleni Frangou, Dimitrios Goumenos, Marten Segelmark, Kultigin Turkmen, Cees van Kooten, Vladimir Tesar, Duvuru Geetha, Fernando C Fervenza, David R W Jayne, Kate I Stevens, Andreas Kronbichler

**Affiliations:** Division of Pulmonary and Critical Care Medicine, Department of Medicine, Mayo Clinic College of Medicine and Science, Rochester, MN, USA; Faculdade de Medicina da Universidade do Porto, Departamento de Biomedicina, Porto, Portugal; Department of Internal Medicine IV (Nephrology and Hypertension), Medical University Innsbruck, Innsbruck, Austria; Department of Internal Medicine IV, Hospital of the Ludwig Maximilians University, Munich, Germany; Department of Health, Medicine and Caring Sciences, Linköping University, Linköping, Sweden; Department of Renal Medicine, Karolinska University Hospital and CLINTEC Karolinska Institutet, Stockholm, Sweden; Department of Nephrology, Hospital Universitario La Paz, IdiPAZ, Madrid, Spain; Division of Nephrology, RWTH Aachen University Hospital, Aachen, Germany; Department of Nephrology, Limassol General Hospital, SHSO, Cyprus; Medical School, University of Nicosia, Nicosia, Cyprus; Department of Nephrology and Renal Transplantation, Patras University Hospital, Patras, Greece; Division of Nephrology, Department of Clinical Sciences Lund, Lund University and Skane University Hospital, Lund, Sweden; Division of Nephrology, Department of Internal Medicine, Necmettin Erbakan University, Konya, Turkey; Division of Nephrology and Transplant Medicine, Department of Medicine, Leiden University Medical Center, Leiden, The Netherlands; Department of Nephrology, First Faculty of Medicine and General University Hospital, Charles University, Prague, Czechia; Division of Nephrology, Department of Medicine, Johns Hopkins University School of Medicine, Baltimore, MD, United States of America; Division of Nephrology and Hypertension, Department of Medicine, Mayo Clinic College of Medicine and Science, Rochester, MN, USA; Department of Medicine, University of Cambridge, Cambridge, United Kingdom; Division of Nephrology and Transplantation, Queen Elizabeth University Hospital, Glasgow, United Kingdom; Department of Medicine, University of Cambridge, Cambridge, United Kingdom

**Keywords:** ANCA-associated vasculitis, glomerulonephritis, granulomatosis with polyangiitis, guidelines, microscopic polyangiitis

## Abstract

Updated guidelines on the management of antineutrophil cytoplasmic antibody (ANCA)-associated vasculitis (AAV) were released in 2021 by the American College of Rheumatology jointly with the Vasculitis Foundation and, subsequently, in 2022 by the European Alliance of Associations for Rheumatology. In addition, in 2021, the Kidney Disease: Improving Global Outcomes had released updated recommendations on the treatment of AAV with glomerulonephritis (AAV-GN). Kidney involvement is particularly relevant in microscopic polyangiitis and granulomatosis with polyangiitis, but is less frequent in eosinophilic granulomatosis with polyangiitis. The management of AAV-GN has been a focus for drug development and change over the past 10 years. Avoidance of progression to end-stage kidney disease (ESKD) or kidney failure is one of the main unmet needs in the management of AAV, with ESKD having a major impact on morbidity, health costs and mortality risk. Relevant changes in AAV-GN management are related to remission-induction treatment of patients with severe kidney disease, the use of glucocorticoids and avacopan, and remission-maintenance treatment. All the documents provide guidance in accordance with the evidence-based standard of care available at the time of their release. With our work we aim to (i) show the progress made and identify the differences between guidelines and recommendations, (ii) discuss the supporting rationale for those, and (iii) identify gaps in knowledge that could benefit from additional research and should be revised in subsequent updates.


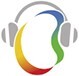
 Watch the video of this contribution at https://academic.oup.com/ndt/pages/author_videos

## INTRODUCTION

Kidney involvement in antineutrophil cytoplasmic antibody (ANCA)-associated vasculitis (AAV) has an important impact on survival and long-term prognosis [1–3]. AAV glomerulonephritis (AAV-GN) is frequent in microscopic polyangiitis (MPA) and granulomatosis with polyangiitis (GPA), and confers higher morbidity and mortality [4–8]. Progression to end-stage kidney disease (ESKD) or kidney failure is declining but it is still an unmet need in the management of AAV, particularly in organ-threatening presentations [8–14]. Thus, early diagnosis and adequate treatment of kidney involvement are crucial for the outcomes in AAV.

The American College of Rheumatology (ACR) jointly with the Vasculitis Foundation (VF) released recommendations together for the first time in 2021 [15]. The Voting Panel was formed to develop recommendations for the seven forms of systemic vasculitis, including AAV. The guidelines followed the ACR guideline development process which uses the GRADE (Grading of Recommendations Assessment, Development and Evaluation) methodology to rate the quality of the evidence [16, 17].

The European Alliance of Associations for Rheumatology (EULAR) updated their recommendations in 2022 [18]. Since publication of the ACR/VF recommendations, relevant literature with potential impact for AAV-GN management has been published, particularly the landmark Avacopan for the Treatment of ANCA-Associated Vasculitis (ADVOCATE) trial and updated meta-analysis concerning potential indications for plasma exchange (PLEX) use.

The Kidney Disease: Improving Global Outcomes (KDIGO) released recommendations for the management of GN, including AAV-GN, in 2012 [19], followed by a report from the 2017 Controversy Conference [20], and finally a full updated document in 2021 [21]. The KDIGO recommendations used the GRADE approach to rate the quality of the evidence and the strength of the recommendations. Practice points were added when there were no systematic reviews, insufficient or inconclusive evidence or illogical alternatives.

The Immunonephrology Working Group (IWG) of the European Renal Association (ERA) reviewed the three documents to determine the common principles of AAV treatment, unique aspects of kidney involvement management, differences in recommendations and gaps in the knowledge that apply to patients with AAV-GN, particularly patients with MPA and GPA. Due to the low incidence of kidney involvement in eosinophilic GPA (EGPA) and the fact that patients with EGPA are not included in many of the trials, considerations on patients with EGPA are outside the scope of this paper. Additional information on the respective guideline creation processes and panel compositions together with an overview of general principles for the management of GN as addressed in the KDIGO 2021 recommendations are provided in the Supplementary Appendix.

## GENERAL PRINCIPLES OF AAV MANAGEMENT

Some overarching principles are common to the ACR/VF guidelines and the EULAR and the KDIGO recommendations. Treatment with rituximab (RTX) has emerged as the first line for remission-induction treatment in the ACR/VF guidelines, while EULAR recommendations and the KDIGO guideline favor the use of RTX or cyclophosphamide (CYC), which depends upon factors outlined in Table 1 [15, 18, 21].

**Table 1:** Factors that support the choice of RTX over CYC (and vice versa) for remission-induction treatment in AAV.

RTX favored	Children and adolescents
	Pre-menopausal women and men concerned about their fertility
	Frail older adults
	Glucocorticoid-sparing
	Relapsing disease
	PR3-ANCA
CYC favored	Difficult access to RTX
	The efficacy of combining CYC and RTX for the treatment of patients with SCr >350 µmol/L is under study (NCT03942887) but it has been historically considered as a possible or favorable option

Further details on general remission-induction and remission-maintenance treatment and prophylaxis of infections and treatment of relapsing AAV are provided in the Supplementary Appendix.

## AAV-GN MANAGEMENT

**Figure 1:**
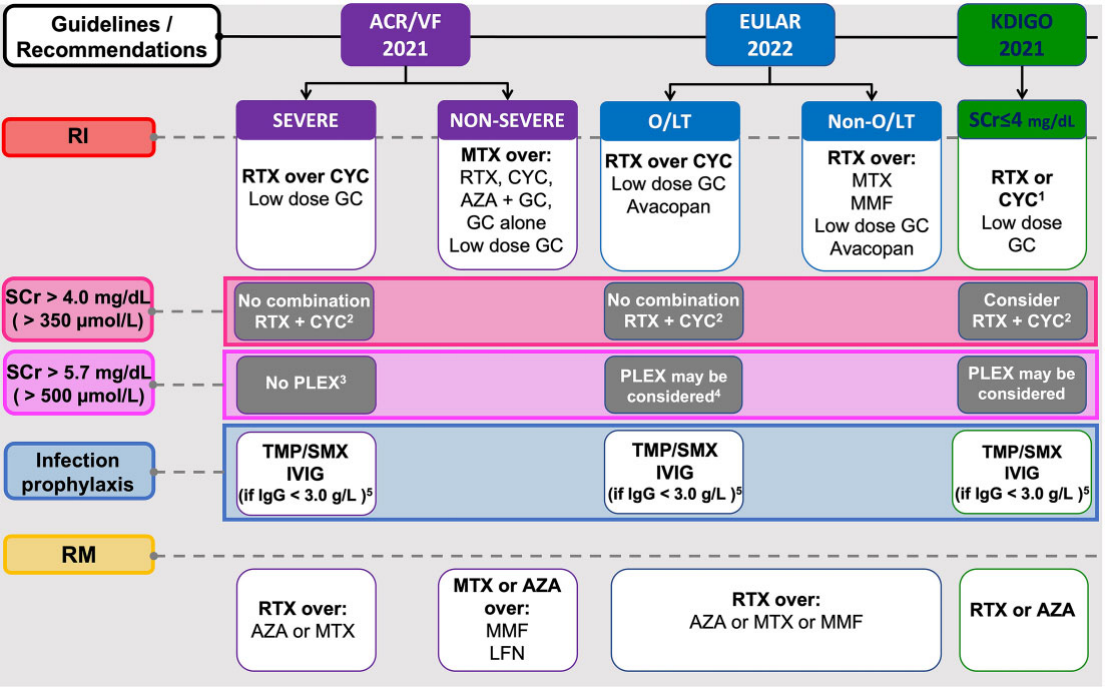
Comparison of the ACR/VF guidelines, the EULAR recommendations and KDIGO guideline on AAV management. (1) According to individual factors (Table 2). (2) No RCTs have assessed the benefit of RTX/CYC combination over RTX. However, the RTX/CYC combination has been shown to reduce cumulative CYC exposure in RITUXVAS and retrospective studies have indicated the possibility of GC minimization and improved responses that require investigation in an RCT (NCT03942887). (3) The ACR/VCRC guidelines recommend against the routine use of PLEX. However, it is added that PLEX may be considered for certain patients with active GN or those who are critically ill and whose disease is not responding to recommended remission induction therapies (i.e. plasma exchange as “salvage” or “rescue” therapy). Thus, there is no reference to the severity of kidney disease. (4) PLEX may be considered as part of therapy to induce remission in GPA or MPA for those with a SCr >300 µmol/L (>3.4 mg/dL) due to active GN. Routine use of plasma exchange to treat alveolar hemorrhage in GPA and MPA is not recommended. (5) IVIG or immunoglobulin G replacement therapy should be considered in the context of severe infection and hypogammaglobulinemia. IVIG, intravenous immunoglobulin; LFN, leflunomide; O/LT, organ-/life-threatening; RI, remission-induction; RM, remission-maintenance; TMP/SMX, trimethoprim/sulfamethoxazole.

When discussing different guidelines and recommendations, the terminology and use of organ-/life-threatening disease versus severe and limited disease further complicates direct comparison (Table 2). We analyzed all the documents and either found differences in AAV-GN management recommendations or highlighted topics worthy of discussion. Those are mainly related to remission-induction treatment in patients with severe kidney disease, the use of glucocorticoids (GC) and avacopan and remission-maintenance treatment options (Fig. 1, Table 3). We summarize our main findings in Box 1.

**Table 2:** Examples of organ-/life-threatening vs not organ-/life-threatening manifestations in AAV.

Organ-/life-threatening manifestations^a^	Not organ-/life-threatening manifestations^b^
GN	Nasal and paranasal disease without bony involvement (erosion) or cartilage collapse or olfactory dysfunction or deafness
Pulmonary hemorrhage	Skin involvement without ulceration
Meningeal involvement	Myositis (skeletal muscle only)
Central nervous system involvement	Non-cavitating pulmonary nodules
Retro-orbital disease	Episcleritis
Cardiac involvement	
Mesenteric involvement	
Mononeuritis multiplex	
+ Limb/digit ischemia (ACR/VF)	

^a,b^The nomenclature regarding the severity of AAV manifestations is evolving. Grossly, “severe” manifestations as defined by the BVAS and used in the ACR/VF guidelines correspond to organ-/life-threatening manifestations as used in the EULAR recommendations, whereas the “non-severe” correspond to the not organ-/life-threatening manifestations.

**Table 3:** Differences in recommendation topics with implication in the treatment of AAV-GN.

Remission induction treatment	ACR/VF [15]	EULAR [18]	KDIGO [21]	Data
Organ-/life-threatening (“severe”) kidney involvementSCr >4 mg/dL				
(i) RTX vs CYC	It remains controversial whether CYC should be preferred in patients with acute kidney injury (SCr >4 mg/dL)Evaluation for kidney transplant in patients in ESKD	RTX or CYC + GC; RTX preferred in relapsing disease	Not enough evidence for the remission-induction treatment with RTXCYC + GC preferred (RTX + CYC may be considered)Discontinue immunosuppressive therapy after 3 months in patients who remain on dialysis and who do not have extrarenal manifestations	*Post hoc* analysis RAVE trial [22]: 18 vs 14 with eGFR <30 mL/min/1.73 m^2^Shah *et al*. [25]: 14 patients received RTX + GC ± PLEX (eGFR <20 mL/min/1.73 m^2^)Geetha *et al*. [26]: 12 patients received RTX + GC vs 25 patients received RTX + CYC + GC (eGFR <20 mL/min/1.73 m^2^)Casal Moura *et al*. [27]: 64 received RTX vs 161 received CYC (propensity score analysis adjusted for eGFR <15 mL/min/1.73 m^2^)
(ii) RTX + CYC	Evidence for combined CYC + RTX therapy for remission-induction remain limited and it is potentially associated with toxicity	No evidence from RCTs to support the combination or RTX and CYC (retrospective data support the use in severe cases)	Combination of 4 weekly infusions of RTX and two pulses of IV CYC with GC might be an alternative to IV CYC for 3–6 monthsRTX 375 mg/m^2^/week ×4 weeks + IV CYC 10 mg/kg at weeks 0 and 2 *or*RTX 1 g at 0 and 2 weeks with CYC 500 mg/m^2^ weeks ×6	RITUXVAS trial [23]ENDURRANCE-1 [33]Shah *et al*. [25]: 14 patients received RTX + GC ± PLEX (eGFR <20 mL/min/1.73 m^2^)Geetha *et al*. [26]: 12 patients received RTX + GC vs 25 patients received RTX + CYC + GC (eGFR <20 mL/min/1.73m^2^)
				Mansfield *et al*. [28]: RTX 1 g at Day 0 and 14 + IV CYC at Day 0 and then every 14 days for a total of six dosesGulati *et al*. [29]: low-dose pulsed IV CYC + RTX + PLEXPepper *et al*. [30]: RTX ×2 + 3 months of low-dose CYC + short course of oral GC (for between 1 and 2 weeks)Cortazar *et al*. [31]: RTX + 2-month oral, low-dose CYC + accelerated PDN taper
(iii) PLEX vs no-PLEX	Against the routine addition of PLEX with active GN or DAH PLEX may be considered for certain patients with active GN or critically ill patients which are not responding to treatmentPLEX is indicated in AAV-GN + anti-GBM antibodies	PLEX may be considered in patients with a SCr >300 µmol/LPLEX is indicated in AAV-GN + anti-GBM antibodies7 treatments over max. 14 days, 60 mL/kg volume replacement, albumin substitution (with FFP in case of bleeding)	Consider PLEX in patients with SCr >5.7 mg/dL or rapidly increasing SCr and in patients with DAH who have hypoxemiaPLEX is indicated in AAV-GN + anti-GBM antibodies	MEPEX trial [34]PEXIVAS trial [35]Walsh *et al*. [36]Zeng *et al*. [37]Yamada *et al*. [74]Casal Moura *et al*. [27]: 51 received PLEX vs 200 did not receive PLEX (propensity score analysis adjusted for eGFR <15 mL/min/1.73 m^2^)
Non-life threatening (“non-severe”) kidney involvement				
(i) MMF	Last line of treatment for non-severe GPA	MMF is a second-line therapy for GPA or MPA	Consider MMF if no organ-threatening involvementPreference in MPO-AAV (over PR3)2000 mg/day (divided doses) with increase to 3000 mg/day for poor response treatment	MYCYC trial [46]Tuin *et al*. [47]Berti *et al*. [48]
Glucocorticoids and glucocorticoid-sparing strategies				
(i) GC use	IV pulse or high-dose oral GC may be prescribedReduced GC regimen over a standard-dose GC regimen for remission-induction	GC at a starting dose of 50–75 mg of prednisolone-equivalent a day (depending on body weight) as used as the reduced GC dose protocol of PEXIVAS; achieving a dose of 5 mg prednisolone-equivalent per day by 4–5 months; no recommendation to use IV methylprednisolone	IV methylprednisolone 1–3 g total dose (possible; without explicit recommendation)Reduced GC dose protocol of PEXIVAS trial is recommendedOral prednisone 1 mg/kg/dayTapering adapted to initial absolute dose/body weight. For CYC induction tapering to 5 mg/day by 6 months, for RTX induction withdrawal by Month 6	PEXIVAS trial [35]LoVAS trial [51]: prednisolone 0.5 mg/kg/day + RTX 375 mg/m^2^/week ×4Chanouzas *et al*. [50]
(ii) Avacopan	Not approved by FDA at the time of the last literature search	Alternative to standard GC; use in combination with low-dose GC regimen, according to the label30 mg twice daily	No formal recommendation	ADVOCATE trial [52]van Leeuwen *et al*. [55]Gabilan *et al*. [56]
Remission-maintenance treatment				
(i) RTX vs AZA	RTX is recommended in severe GPA/MPA: 500 mg every 6 months (FDA approved); 1000 mg every 4 months; 1000 mg every 6 monthsFollowing the same remission-induction regimen for maintenance is recommended in non-severe GPA/MPANo TMP/SMX	RTX is recommended: 500 mg every 6 months (FDA approved); 1000 mg every 4 months; 1000 mg every 6 monthsNo TMP/SMX	RTX or AZARTX: 500 mg every 6 months (FDA approved); 1000 mg every 4 monthsAZA: 1.5–2 mg/kg/day at complete remission until 1 year after diagnosis then decrease by 25 mg every 3 monthsNo TMP/SMX	MAINRITSAN trial [58]RITAZAREM trial [59]
(ii) Scheduled re-dosing vs CD19^+^ B-cell	Scheduled	Scheduled	Scheduled	MAINRITSAN 2 trial [60]
(iii) Optimal length of therapy	RTX ≥18 months	24–48 months, potentially longer depending on preferences	AZA + low GC = 18–48 monthsRTX = 18 months	MAINRITSAN 3 trial [63]REMAIN trial [62]AZA-ANCA trial [64]
(iv) No start/withdrawalMonitoring disease activity	Not addressedANCA levels are not recommended	Not addressedANCA levels are not recommended	No consensus(i) Patients that remain in ESKD after remission-induction do not need to be started on maintenance treatment(ii) Patients with MPO-AAV after achieving remission with RTX could avoid remission-maintenance treatment due to the low frequency of relapses if they can be adequately monitoredANCA levels are not recommended	MASTER-ANCA trial (ongoing)Casal Moura *et al*. [65]Kemna *et al*. [69]Fussner *et al*. [70]

IVIG, intravenous immunoglobulin; LFN, leflunomide; TMP/SMX, trimethoprim/sulfamethoxazole; GBM, glomerular basement membrane FFP, fresh frozen plasma; ENDURRANCE-1, ExploriNg DUrable Remission with Rituximab in ANCA-associatEd vasculitis; MYCYC, Mycophenolate mofetil versus cyclophosphamide for remission induction in ANCA-associated vasculitis, LoVAS, Low-dose Glucocorticoid Vasculitis Induction Study; MAINRITSAN, Rituximab versus Azathioprine for Maintenance in ANCA-Associated Vasculitis; RITAZAREM, Rituximab versus azathioprine for maintenance of remission for patients with ANCA-associated vasculitis and relapsing disease; REMAIN, Randomised controlled trial of prolonged treatment in the remission phase of ANCA-associated vasculitis; AZA-ANCA, Prevention of Relapses in Proteinase 3 (PR3)-Anti-neutrophil Cytoplasmic Antibodies (ANCA)-Associated Vasculitis; MASTER-ANCA, Maintaining or Stopping Immunosuppressive Therapy in Patients With ANCA Vasculitis and End-stage Renal Disease; PDN, prednisone.

**Box 1. Take-home messages obtained from the guideline comparison.**

Remission-induction treatment in patients with organ-/life-threatening kidney disease

- RTX vs CYC: the lack of consensus in the international community remains because there is no prospective evidence to support the use of RTX for remission-induction in severely impaired kidney disease, despite the increasing use of RTX in this patient cohort.
- RTX and CYC: the safety of this combination is currently being tested in ongoing clinical trials and an additional benefit needs to be shown prior to recommending extensive use.
- PLEX vs no-PLEX: the clinical practice is heterogenous and guidelines/recommendations differences reflect differences in clinical practice in the USA vs Europe. However, the addition of PLEX for severe kidney disease may be contemplated.

Remission-induction treatment in patients with non-organ-/life-threatening kidney involvement

- MMF: there is evidence that MMF is a reasonable option in patients with non-severe kidney involvement, but its use should be limited to MPO-ANCA patients and mainly considered in the presence of contraindications/economic constraints to other remission-induction treatments.

Glucocorticoids use, glucocorticoid sparing and avacopan

- Glucocorticoids: reduction of exposure to GC is an attainable goal without compromising the efficacy of remission-induction treatment while reducing the frequency of side effects. This practice should be introduced in the clinical management of these patients.
- Avacopan: the adjunct use of avacopan is effective and safe, with promising effects on kidney recovery and, thus, its use should be encouraged particularly in patients with severe kidney involvement.

Remission-maintenance treatment

- RTX vs AZA: RTX is the first-line therapy for remission-maintenance. AZA or MTX (in patients with eGFR >60 mL/min/1.73 m^2^) or MMF are alternatives in the appropriate context.
- RTX use guided by fixed intervals vs CD19^+^ B-cell reconstitution: RTX re-dosing in fixed intervals is favored.
- Optimal length of non-GC immunosuppressive remission-maintenance treatment: a minimal period of 18 months of remission-maintenance treatment is recommended, although longer periods of treatment were suggested to decrease the risk of relapse. Additionally, the optimal duration of treatment might be different for MPO- vs PR3-ANCA patients due to the risk of relapse impacting their monitoring strategies and ANCA status during remission.
- No start or withdrawal: more studies are needed to establish a position.

Monitoring disease activity

- Monitoring strategies based on ANCA should take ANCA specificity and organ involvement into consideration and might particularly suit patients with MPO-AAV-GN.

## REMISSION-INDUCTION TREATMENT IN PATIENTS WITH ORGAN-/LIFE-THREATENING KIDNEY DISEASE

Recommendations for the optimal remission-induction treatment strategy in patients with AAV-GN and severely reduced or rapidly declining estimated glomerular filtration rate (eGFR) (<15.0 mL/min/1.73 m^2^) differ. This is mainly due to the systematic exclusion of patients with serum creatinine (SCr) >4 mg/dL from the remission-induction clinical trial in which RTX was directly compared with CYC alone.

### RTX vs CYC

In the KDIGO guideline, the panel clearly considered that the evidence on the use of RTX for remission-induction is limited in patients with SCr >4 mg/L, favoring treatment with CYC and GC for 3–6 months [21]. In the ACR/VF guidelines, the panel concluded that preferential use of CYC in these patients remains controversial [15]. The EULAR recommendations follow the example of KDIGO and suggest a remission-induction therapy with either RTX or CYC [18]. Only a subset of patients of 32 patients (18 on the RTX arm and 14 on the CYC) with an eGFR <30.0 mL/min/1.73 m^2^ at baseline was included in the Rituximab versus Cyclophosphamide for ANCA-Associated Vasculitis (RAVE) trial [22]. The response to remission-induction treatment was similar [23]. In the Rituximab versus Cyclophosphamide in ANCA-Associated Renal Vasculitis (RITUXVAS) trial, there was no head-to-head comparison since the test group patients received RTX and CYC [24]. Several observational studies tried to address this question [25–27]. In patients with eGFR <20.0 mL/min/1.73 m^2^, two studies showed that there were no differences in remission, renal recovery from ESKD or death when patients were treated with RTX and GC [25, 26]. In addition, a propensity-score matching analysis adjusted for eGFR <15.0 mL/min/1.73 m^2^ and compared patients who received RTX or CYC for remission-induction (60 vs 60 patients) [27]. There were no differences in the frequency of remission or risk for renal events between groups [27].

#### Summary

The lack of consensus in the international community remains because there is no prospective evidence to support the use of RTX for remission-induction in severely impaired kidney disease, despite the increasing use of RTX in this patient cohort.

### RTX and CYC

The combination of RTX with CYC is not endorsed by the ACR/VF guidelines due to limited data and potential toxicities [15]. In contrast, the panel from KDIGO considered that the combination of 4-weekly infusions of RTX with two pulses of intravenous (IV) CYC and GC might be an alternative to IV CYC for 3–6 months [21]. The EULAR recommendations point towards an ongoing randomized controlled trial (RCT) investigating a combination of RTX and CYC, but otherwise refer to the retrospective nature of evidence [18]. Combined regimes of CYC and RTX have emerged not only in the context of the treatment of severe and refractory disease but also to reduce the cumulative doses of both CYC and GC [24, 28–31]. The CYCLowVasc study showed that the combination of RTX with low-dose CYC was effective at inducing long-term disease-free remission in patients with severe kidney disease [28]. McAdoo *et al.* [32] have shown that a combination of oral GC, 2 g of RTX and median of 3 g of CYC followed by a maintenance therapy with azathioprine (AZA) in 66 patients with AAV-GN was superior in terms of survival, ESKD rate and relapse when compared with 198 propensity-matched cases from EUVAS trials. Notably, the EUVAS trials were conducted at least a decade before this retrospective study was initiated, potentially influencing the results of this analysis. Reduction in the rate of ESKD at 36 months with low-dose CYC and GC regimen and two doses of RTX and PLEX was recently reported [29]. The Rituximab/Cyclophosphamide and Minimal Dose Glucocorticoid in AAV (SMARTVAS) study showed that patients who received a combined regimen of two doses of RTX, 3 months of low-dose CYC, and a short course of GCs had a substantial reduction of the CYC and GC doses [30]. Fewer GC-related adverse events, namely severe infections and diabetes, were reported [30]. Data from ongoing clinical trials (NCT03942887) are awaited [33]. However, previous studies have shown that RTX alone was not inferior to the combination of RTX and CYC in this population [25, 26], thus an additional benefit needs to be shown.

#### Summary

The safety of this combination is currently being tested in ongoing clinical trials and an additional benefit needs to be shown prior to recommending extensive use.

### PLEX vs no-PLEX

The ACR/VF panel conditionally recommended against the routine addition of PLEX with active GN or diffuse alveolar hemorrhage (DAH) but they add that PLEX may be considered for certain patients with active GN or who are critically ill and are not responding to treatment [15]. KDIGO included a practice point to consider PLEX in patients with SCr >5.7 mg/dL (500 µmol/L) or rapidly increasing SCr despite treatment and in patients with DAH who have hypoxemia [21]. The EULAR recommendations state that PLEX may be considered in patients with a SCr >3.4 mg/dL (300 µmol/L) [18]. After the publication of the Plasma Exchange for Renal Vasculitis (MEPEX) trial in 2007, PLEX in addition to remission-induction therapy became the standard of care for patients with AAV and severe kidney disease (SCr >5.7 mg/dL) [34]. The Plasma Exchange and Glucocorticoids in Severe ANCA-Associated Vasculitis (PEXIVAS) trial aimed to expand the indication for PLEX for patients with a preserved kidney function. However, the trial failed to show a benefit in the composite endpoint of death or ESKD (hazard ratio 0.86; 95% confidence interval 0.65–1.13; *P* = .27) when compared with standard remission-induction treatment alone [35]. This was also observed in the sub-analysis of patients with SCr >5.6 mg/dL and alveolar hemorrhage [35]. The 2022 EULAR recommendations echo a recent meta-analysis of all studies, over a 40-year-period, in which PLEX was used for the treatment of AAV. Whilst there was no effect of PLEX on mortality, a reduced risk of ESKD at 1 year at the cost of an increased risk of serious infections was observed [36]. A rapid recommendation favoring the addition of PLEX to remission-induction treatment in patients with a SCr >300 µmol/L followed [37]. However, the conclusions of this meta-analysis have been debated [38]. Nevertheless, PLEX is used by 40% of surveyed physicians especially in the setting of a SCr of >5.7 mg/dL, with predominant use amongst nephrologists rather than rheumatologists [39].

In propensity-score matching analysis, adjusted for eGFR <15.0 mL/min/1.73 m^2^ and comparing patients who received PLEX vs those who did not (46 vs 46 patients), the addition of PLEX had no benefit on remission, renal events or mortality [27]. Another retrospective study of 188 patients with AAV and severe kidney disease found no efficacy of PLEX on death or ESKD [40]. Finally, another study on the efficacy of PLEX in patients with eGFR <15.0 mL/min/1.73 m^2^ showed short-term benefits with higher dialysis-free rate at 12 months in patients treated with CYC [41]. Similarly, strong evidence of PLEX in DAH is lacking due to small numbers in PEXIVAS, with available studies showing no statistical difference in mortality in patients with hypoxemia [42].

#### Summary

The clinical practice is heterogenous and guidelines/recommendations differences reflect differences in clinical practice in the USA vs Europe. However, the addition of PLEX for severe kidney disease may be contemplated.

## REMISSION-INDUCTION TREATMENT IN PATIENTS WITH NON-ORGAN-/LIFE-THREATENING KIDNEY INVOLVEMENT

The primary goal is to minimize the use of potent immunosuppression while successfully inducing remission in patients with non-severe AAV-GN. Avoiding relapse has posed the main challenge.

### MMF

In the KDIGO algorithm for AAV-GN treatment, mycophenolate mofetil (MMF) can be considered in patients without organ-threatening involvement [21]. However, in the ACR/VF guidelines, MMF is considered the last line of treatment for patients with GPA and there is no mention of patients with non-severe kidney involvement [15]. In the EULAR recommendations, MMF can be considered as a second-line therapy [18]. MMF efficacy in patients with non-severe kidney disease has been demonstrated in clinical trials [43–47]. MMF was associated with higher sustained remission rates in patients with AAV-GN included in clinical trials [48], but concerns regarding the increased rates of relapse when compared with CYC have been raised [46, 47]. MYCYC, an RCT comparing MMF with CYC showed non-inferiority of MMF in inducing remission at 6 months although relapses occurred more frequently in patients with proteinase 3 (PR3)-ANCA vasculitis treated with MMF [46]. A recent meta-analysis underlines that MMF may play a particular role in patients with myeloperoxidase (MPO)-ANCA with mild to moderate kidney involvement [49].

#### Summary

There is evidence that MMF is a reasonable option in patients with non-severe kidney involvement, but its use should be limited to MPO-ANCA patients and mainly considered in the presence of contraindications/economic constraints to other remission-induction treatments.

## GLUCOCORTICOIDS USE, GLUCOCORTICOID SPARING AND AVACOPAN

The addition of high dose of GC (methylprednisolone IV pulses of 500–1000 mg/day and 1–3 g total dose or high doses of oral prednisone, 1 mg/kg/day) to induce remission is a common practice despite the absence of direct clinical trials, and GC confer a high side effect burden which should be mitigated.

### Glucocorticoids

In KDIGO, GC are considered part of the remission-induction therapy and support the adoption of the reduced corticosteroid dose used in the PEXIVAS trial [21, 35]. The ACR/VF panel adopted an ungraded position regarding the use of IV pulse GC or high-dose oral GC, but conditionally recommend a reduced GC oral regimen over a standard-dose GC oral regimen for remission-induction [15]. The EULAR recommendations also recommend the reduced GC oral regimen as assessed in the PEXIVAS trial, and stress that there is no compelling evidence to use IV methylprednisolone pulses to induce remission [18]. The need for IV methylprednisolone pulses has been questioned [50] and the avoidance of methylprednisolone pulses has reduced the occurrence of diabetes and severe infections without compromising efficacy in a retrospective study [50]. The PEXIVAS trial provided strong evidence for an efficient and safe reduced oral GC tapering regimen, although the majority of patients in this trial received CYC and there remain concerns about reduced oral GC protocols with rituximab induction [35]. This was also verified in the sub-analysis of patients with severe disease (SCr >5.6 mg/dL and DAH with severe hypoxemia) [35]. The LoVAS trial also showed that, in patients with non-severe disease, low dose of prednisolone (0.5 mg/kg/day with RTX 375 mg/m^2^/week ×4) was non inferior to high-dose prednisolone (1 mg/kg/day with RTX) [51]. This trial has mainly recruited older, MPO-ANCA patients from Japan with preserved kidney function, hampering generalizability. However, these results show that reduction of GC is a feasible and efficient way to reduce treatment-associated morbidity in AAV.

#### Summary

Reduction of exposure to GC is an attainable goal without compromising the efficacy of remission-induction treatment while reducing the frequency of side effects. This practice should be introduced in the clinical management of these patients.

### Avacopan

In the ACR/VF guidelines and in the KDIGO guideline there are no specific statements regarding the use of avacopan since it was not approved by Food and Drug Administration (FDA) at the time of literature search [15, 21, 52]. The KDIGO guidelines acknowledge the option of complement targeted therapy as another strategy to potentially reduce GC exposure [21]. The EULAR recommendations do consider a role for avacopan, for induction of remission in GPA or MPA as a strategy to reduce exposure to GC [18].

The alternative complement pathway is relevant in the pathophysiology of AAV [53] and the ADVOCATE trial studied oral avacopan, a small molecule inhibiting the C5aR1, compared with oral prednisone on a tapering schedule in combination with either CYC (followed by AZA) or RTX. Avacopan treatment was noninferior at 26 weeks for inducing remission and superior at 52 weeks for sustained remission [52]. Avacopan treatment showed advantages in secondary outcomes, e.g. reduced relapse rate within the first 52 weeks and a higher eGFR recovery at Week 26 and 52 when compared with GC tapering [52]. Cortazar *et al.* [54] recently showed that among patients with baseline eGFR ≤20 mL/min/1.73 m^2^ in the ADVOCATE trial, eGFR improved more in the avacopan group when compared with the prednisone group (*P* = .030). Of note, GC use was lower but not absent in avacopan- compared with GC-treated patients (1348.9 ± 2040.29 mg versus 3654.5 ± 1709.83 mg mean total dose ± SD, respectively). RTX was not approved for maintenance treatment at the time that ADVOCATE was designed and patients treated with RTX for remission induction in this trial did not receive further maintenance immunosuppression, leading to an imbalance between the two arms, as RTX-treated patients in the prednisone arm were off immunosuppression from Week 21. Meanwhile, small case series using avacopan in a compassionate-use program for selected patients with refractory disease or contraindication for steroid use were published [55, 56]. While avacopan seems a promising option to reduce GC in AAV, uncertainties regarding optimal duration of treatment (e.g. when combined with RTX maintenance treatment), the residual need for GC, long-term follow-up, and the effect in patients with eGFR <15 mL/min/1.73 m^2^ [52].

#### Summary

The adjunct use of avacopan is effective and safe, with promising effects on kidney recovery and, thus, its use should be encouraged particularly in patients with severe kidney involvement.

## REMISSION-MAINTENANCE TREATMENT

Remission-maintenance treatment is established to prevent relapse and avoid organ damage progression [57]. The main challenge has been to tailor the remission-maintenance regimen to the patients’ needs and determine the optimal duration of therapy.

### RTX vs AZA

All the panels agree that remission-maintenance treatment should be given to patients with AAV following remission-induction treatment. RTX is the treatment of choice for patients with severe GPA/MPA (and thus AAV-GN) according to the ACR/VF guidelines and EULAR recommendations [15, 18]. In contrast, in the KDIGO guideline, patients might start remission-maintenance treatment with either RTX (without GC) or AZA in association with low GC dose [21]. Nevertheless, the panel felt that the evidence is weighted towards the use of RTX [21]. In severe GPA/MPA, RTX has the best evidence for the maintenance of remission when compared with AZA [58, 59]. In the MAINRITSAN trial, the authors showed lower relapse rates at 28 months in the RTX arm (500 mg on Days 0 and 14, and Months 6, 12 and 18) when compared with AZA [58]. The RITAZAREM trial showed that RTX was efficient in reinducing and maintaining remission in patients with relapsing GPA/MPA [59]. AZA or methotrexate (MTX) (in patients with eGFR >60 mL/min/1.73 m^2^) or MMF are viable alternatives for patients in which RTX cannot be used, or in patients with non-severe GPA/MPA [15]. While KDIGO mention a meta-analysis indicating that a longer course of GC may be associated with fewer relapses [49], all the panels agree that GC tapering should occur as early as possible, accepting that a small number of patients will remain on a low GC dose longer-term. The best strategy for GC tapering in remission-maintenance is under review in The Assessment of Prednisone in Remission Trial (TAPIR) and MAIPENSAN studies.

#### Summary

RTX is the first-line therapy for remission-maintenance. AZA or MTX (in patients with eGFR >60 mL/min/1.73 m^2^) or MMF are alternatives in the appropriate context.

### RTX use guided by fixed intervals vs CD19^+^ B-cell reconstitution

The panels from ACR/VF, EULAR and KDIGO consider that RTX remission-maintenance re-dosing should be scheduled at fixed intervals [15, 18]. The MAINRITSAN2 trial showed no differences between a scheduled regimen or a regimen guided by CD19^+^ B-cell reconstitution, but this study was underpowered [60]. However, RTX re-dosing guided by CD19^+^ B-cell reconstitution resulted in fewer infusions as initially described in observational studies [60, 61]. It is unclear whether CD19^+^ B-cell reconstitution and ANCA serum levels could be informative in populations with low risk of relapse, such as MPO-ANCA patients with AAV-GN.

#### Summary

RTX re-dosing in fixed intervals is favored.

### Optimal length of non-GC immunosuppressive remission-maintenance treatment

The ACR/VF panel considered that the optimal duration of remission-maintenance treatment is not well established and stated an ungraded position affirming that the length of treatment should be guided by patients’ clinical condition, preferences and values [15]. In the KDIGO guideline, the optimal duration for AZA + low GC dose is 18–48 months after induction of remission, and for RTX is 18 months after remission [21]. In the EULAR recommendations, a continuation of maintenance therapy for at least 24 months is recommended [18]. Longer durations (up to 48 months) should be considered in relapsing patients or those with an increased risk of relapse, but a fine balance against risks of continuing immunosuppression and patients preferences is needed. The general recommendations are for at least ≥18 months of remission-maintenance and there might be benefit to extend this for longer [62]. Results of the MAINRITSAN3 trial showed that in patients who underwent RTX maintenance treatment and were in sustained remission for 2 years (24 months), an additional 2 years of treatment with RTX decreased the relapse rate (48 months) [63]. Similarly, the tapering of AZA after 48 months was associated with fewer relapses and lower incidence of ESKD, independently of ANCA specificity [62, 64]. This is a particular point where the definition of personalized treatment guided by biomarkers in combination with other factors (including relapse history, extent of organ involvement and ANCA status) is attractive to avoid unnecessary exposure to immunosuppression and some data are available in patients with MPO-AAV-GN [65]. Data specifically characterizing the optimal duration of remission-maintenance with a focus on AAV-GN are lacking. Maintenance of ANCA Vasculitis Remission by Intermittent Rituximab Dosing (MAINTANCAVAS) is a currently recruiting RCT comparing two tailored-RTX strategies for a continued maintenance treatment, using (i) CD19^+^ B-cell reconstitution and (ii) ANCA titers for patients who completed 2 years of maintenance treatment with RTX in fixed intervals (NCT02749292).

#### Summary

A minimal period of 18 months of remission-maintenance treatment is recommended, although longer periods of treatment were suggested to decrease the risk of relapse. Additionally, the optimal duration of treatment might be different for MPO- vs PR3-ANCA patients due to the risk of relapse impacting their monitoring strategies and ANCA status during remission.

### No start or withdrawal

In the KDIGO guideline, there are some considerations regarding patients that remain dialysis-dependent following remission-induction [21]. The panel considered that the institution of remission-maintenance therapy may be unnecessary, particularly in MPO-ANCA patients because relapse rates are low and the risk of infection is high (expert opinion) [66]. The ongoing Maintaining or Stopping Immunosuppressive Therapy in Patients With ANCA Vasculitis and End-stage Renal Disease (MASTER-ANCA) will provide future guidance. Moreover, the panel from KDIGO considered that patients with MPO-AAV who achieve remission after induction with RTX might not need to start remission-maintenance therapy if they can be monitored adequately [21]. This is an expert recommendation based on the low risk of relapse of this subset of patients, and this might be feasible particularly if the ANCA status is also considered (persistent positivity vs negativity vs reappearance) [65, 67].

#### Summary

More studies are needed to establish a position.

## MONITORING DISEASE ACTIVITY

It has been challenging to determine a biomarker that can be used to monitor disease activity. The utility of ANCA serial testing for relapse prediction has been hypothesized. Nevertheless, panels form ACR/VF, EULAR and KDIGO did not recommend monitoring ANCA levels to determine disease activity. This was mainly based on the conclusions from a meta-analysis that included studies in which the relapse risk was assessed in cohorts that combined patients with MPO- and PR3-AAV [68]. Previous studies have shown that a rise in ANCA levels correlated better with relapses in patients with AAV-GN than in patients without renal disease [69, 70]. This strategy might particularly suit patients with AAV-GN and MPO-ANCA [65].

### Summary

Monitoring strategies based on ANCA should take ANCA specificity and organ involvement into consideration and might particularly suit patients with MPO-AAV-GN.

## FUTURE RESEARCH AND GAPS IN KNOWLEDGE

There are several gaps in knowledge where ongoing and future research will have a pivotal role. The lack of robust biomarkers hampers more personalized management of patients with AAV. Nevertheless, biomarkers could be of help to guide treatment decisions especially if response to treatment can be predicted (i.e. optimal dose of RTX, optimal length of remission-maintenance treatment, patients who could benefit from PLEX). However, improved understanding of the pathogenesis might contribute to better tailoring individual risks by integrating the genetic and immunologic information with what we already use to evaluate these patients. Additionally, the new classification criteria will potentially help a more precise selection of patients for inclusion in clinical trials. In particular, the guidelines/recommendations to date do not provide any reference to treatment stratification according to MPO-ANCA and PR3-ANCA specificity. There is cumulative evidence that there are differences in genetics, disease phenotype, relapse rates and response to remission-induction, which could potentially guide initial treatment and the duration of maintenance-remission treatment [57]. However, these findings have not yet been incorporated into clinical trials or clinical practice. Similarly, using kidney biopsy histopathology data might potentially help in risk stratification for the evaluation of the response to remission-induction treatment and PLEX [40, 71–73]. In particular, Nezam *et al.* [40] suggest that a risk prediction model that incorporates clinical and pathological data might identify a subset of patients who benefit from PLEX, and validation is awaited. Clinical trials will continue to help determine how best to use available and new treatments, and in the evaluation of outcomes of interest. It will be interesting to evaluate the performance of avacopan in patients with life-threatening kidney disease: although promising results were recently published by Cortazar *et al.*, these were not available at the time of the EULAR recommendations (presented in June 2022 in Copenhagen), nor were the ADVOCATE trial results published at the time of the publication of the ACR/VCRC and KDIGO guidelines [54–56]. The KDIGO guidelines for ANCA vasculitis are currently undergoing an update.

The scientific community should carefully evaluate results from clinical trials and these should be adequately powered for the research question they are trying to address. In addition, follow-up periods beyond 52 weeks are undoubtedly essential, or even open-label extensions of these trials. Phase 4 trials (such as Rituximab Surveillance Study in Vasculitis (RIVAS) or AVACOSTAR) will inform about the efficacy of these drugs in a real-world clinical practice. Finally, future clinical trial design should use the standard of care as the comparator, e.g. addition of a RTX-based rituximab therapy. Another relevant question is to what extent other type of works (namely retrospective studies) should be utilized for guidelines or recommendations which impact strategies of management of patients with AAV. International collaboration and registries can bolster the knowledge of clinical experience and patient populations, but standardized documentation of patients is a prerequisite for such endeavors. Finally, long-term and follow-up studies are crucial for the understanding of the results of interventions.

## CONCLUSIONS

Early diagnosis of kidney involvement in AAV is crucial to improve short- and long-term renal outcomes in patients at risk of kidney function damage. Management should be initiated as early as possible in experienced centers following the best evidence available. The panels from ACR/VF, EULAR and KDIGO provided expert input that resulted in the generation of guidelines and recommendations that are mainly consistent and timely, and that convey the best evidence-based knowledge available. AAV-GN remains one of the most challenging presentations in patients with AAV. Evidence is lacking or is not compelling due to the difficulties in gathering prospective data in patients with severe kidney disease. Therefore, most of the divergences translate into different practices due to how available evidence is valued and how beliefs shape their application. Our intention is to help to generate a critical appraisal of these documents and stimulate discussions that can propel the research in the field forward.

## Supplementary Material

gfad090_Supplemental_File

## Data Availability

No new data were generated or analysed in support of this research.
