# Supplementary material for: Management of antineutrophil cytoplasmic antibody–associated vasculitis with glomerulonephritis as proposed by the ACR 2021, EULAR 2022 and KDIGO 2021 guidelines/recommendations
Source: Nephrol Dial Transplant. 2023 May 10;38(11):2637–51. doi: 10.1093/ndt/gfad090 (PMC10615627; doi:10.1093/ndt/gfad090)
Supplement: gfad090_Supplemental_File [file gfad090_supplemental_file.docx]

**Supplementary appendix**

**Table of contents**

**S1. Detailed information on guideline creation**

**S2. General principles for the management of GN**

**Supplementary Table 1**

**S3. General principles of AAV management**

**S1. Detailed information on guideline creation and panel composition**

**ACR/VF**

The panel included physicians (rheumatologists) who could provide insights on all forms of vasculitis. The literature review followed a PICO (population, intervention, comparator, and outcome) format question methodology (47 questions for GPA/MPA, and 34 questions for EGPA) and was chaired by a nephrologist. Overall, 26 recommendations and 5 ungraded position statements were outlined for the management of GPA/MPA, and 15 recommendations and 5 ungraded position statements were outlined for EGPA. All recommendations were considered conditional in part due to the lack of multiple randomized controlled trials (RCTs) and/or low-quality evidence. A recommendation was graded as *strong* when supported by moderate to high-quality evidence, and in rare instances based only on very low- to low-certainty evidence. A recommendation *against* was outlined when more harm was expected when compared with very low or low certainty about its benefit.

**EULAR**

The panel consisted of two junior doctors performing the systematic literature review (SLR), which aimed to update the 2016 recommendations, two members of the **Em**erging **EU**LAR **NET**work (EMEUNET), three patient representatives, six nephrologists and thirteen rheumatologists/doctors of medicine. Per EULAR methodology, a convenor led the initiative, and this was performed with the input of a methodologist.

**KDIGO**

A Work Group of experts convened and developed the KDIGO recommendations based on evidence and clinical judgement. The literature review was conducted until 2019 and updated in 2020. A designated Evidence Review Team of multidisciplinary clinical and scientific experts systematically reviewed and analyzed the evidence synthesis of relevant studies.

**S2. General principles for the management of GN**

The general principles of GN management are specifically addressed in the KDIGO 2021 recommendations(20). Some of these principles also apply to AAV-GN management and constitute only practice points, rather than evidence-based recommendations since the studies that underly these are generally of low or moderate quality (Supplementary Table 1). In addition, the management of traditional cardiovascular risk factors (i.e., arterial hypertension, diabetes, hyperlipidemia) should follow specific recommendations.

**Supplementary Table 1 –** General principles of GN treatment with application to the treatment of AAV-GN.

|  | **KDIGO(20)** | **AAV-GN** |
| --- | --- | --- |
| **Kidney biopsy** | Suggested but not absolutely mandatory particularly in the presence of a clinical presentation compatible with the diagnosis of small-vessel vasculitis in combination with proteinase 3 (PR3)- or myeloperoxidase (MPO)-ANCA serology.{, #3486;Aasarod K, 2001 #3533} Treatment start should not be delayed while waiting for biopsy performance or results.(20) | Histological scores show correlation with the severity of kidney dysfunction and with AAV outcomes, providing important prognostic information(22-24).  Yield of 91.5% for the diagnosis in GPA(25). |
| **Kidney function, proteinuria,**  **and hematuria** | The estimation of glomerular filtration rate (GFR) using the race-free Chronic Kidney Disease Collaboration (CKD-EPI) is now the preferred and a well-accepted methodology, particularly in adult patients.(20) In all patients starting immunosuppression, a 24-hour urine collection should be obtained.(20) The presence of hematuria should be routinely assessed by the presence of red cell casts or acanthocytes in urine sediment. | Conflicting evidence considering the correlation of proteinuria and hematuria with AAV disease activity(26-28)..  Hematuria should be periodically monitored for prognostic purposes, namely early detection of relapse(29).  Persisting non glomerular hematuria should prompt urological work-up to exclude hemorrhagic cystitis or bladder cancer (especially after CYC exposure)(30). |
| **Hypertension,**  **proteinuria reduction**  **and dyslipidemia** | Angiotensin-converting enzyme inhibitor (ACEi) and/or angiotensin II receptor blocker (ARB) are first lines therapies in treating patients with both hypertension and proteinuria, and the target systolic blood pressure is <120 mmHg.(20) In patients with persistent dyslipidemia, lifestyle modification including diet, physical activity, weight loss and smoking cessation, are favored.(20) Pharmacologic treatment should be considered according with the with the ASCVD.(20) | Higher risk of developing ASCVD(31, 32). |
| **Infection,**  **vaccination,**  **and prophylaxis** | Pneumococcal and herpes zoster vaccination.  Influenza vaccine should be offered to patients and household contacts.(20)  TMP/SMX should be used as prophylaxis. | No specific recommendations.  Screening for previous/active HBV infection when high immunosuppression (especially rituximab) is considered. Hepatologist consultation & pre-emptive antiviral therapy for HBsAg or anti-HBc positive patients(33). |
| **Diet** | Dietary restriction of sodium to <2 g/day (<90 mmol/day) is important for blood pressure control.(20) Other restriction such as protein and caloric intake should be adjusted to the degree of proteinuria and kidney function, and to achieve normal body mass index, respectively.(20) | No specific recommendations. |
| **Pregnancy and**  **reproductive health** | Women of childbearing age should receive coordinated care between nephrology and obstetrics, with a plan covering pre-pregnancy, antenatal care, delivery, and post-natal care. | Limited data. Successful pregnancies are possible, especially when planned and disease is in remission(34).  Treating active disease is the major challenge. Only few immunosuppressants are considered safe (GC, AZA, CNI). CYC, MTX and MMF are at high risk of fetal harm. Limited data regarding the safety of RTX(35).  Research ongoing (https://www.vasculitisfoundation.org/vpreg/) |
| **Post-transplantation GN** | Assessment of the risk of recurrent disease should be made prior to transplantation but there are no proven strategies to prevent relapse of AAV-GN.(20) | KTx in AAV has similar outcomes than KTx for other causes(36).  Complete remission for ≥6 months before KTx is recommended(20).  Duration of remission or ANCA status before KTx has no influence on relapse rate after KTx(37). |
| **Goals of glomerular**  **disease treatment** | There are few overall goals of treatment that are common to all glomerular diseases: (i) achieving lasting remission; (ii) treatment that avoid or minimize the development of treatment-related adverse events; and (iii) optimize patients’ quality of life while on treatment.(20) | Achieve remission by 3 months. Delayed remission, early relapse and refractory disease are associated with worse outcomes(38). |
|  | | |

**S3. General principles of AAV management**

The ACR/VF based their recommendations on the severity of the disease: for severe GPA/MPA, RTX is conditionally recommended over CYC whereas for non-severe GPA treatment with methotrexate (MTX) is recommended over RTX or CYC, glucocorticoids (GC) alone or azathioprine (AZA) and GC(15). Unfortunately, severity is not robustly defined in any of the guidelines and remains a source of confusion. Likewise, the use of mycophenolate mofetil (MMF) may be considered in patients without organ-threatening involvement according to KDIGO(20). In contrast, EULAR recommendations moved away from those definitions and now recommend remission-induction and remission-maintenance treatment based on the best evidence available. The addition of low dose GC to remission-induction regimens is recommended in all guidelines/recommendations, and a reduced GC dose and schedule according to the PEXIVAS trial is recommended by KDIGO and EULAR(20). Prophylaxis with trimethoprim/sulfamethoxazole (TMP/SMX) in patients receiving RTX or CYC for remission-induction is recommended by ACR/VF guidelines, EULAR and KDIGO recommendations(15).

For remission-maintenance, RTX or AZA are recommended by KDIGO(20). Similarly, to the remission-induction recommendations, the ACR/VF guidelines for remission-maintenance are based on severity of the disease: for severe disease, RTX is recommended over AZA or MTX, and AZA or MTX are recommended over mycophenolate mofetil (MMF), leflunomide (LFN) and TMP/SMX in non-severe disease. EULAR guidelines considered RTX as 1^st^ line maintenance agent, and suggested AZA or MTX as alternatives in special circumstances.

In addition, ACR/VF guidelines recommend *against* adding high dose TMP/SMX to other therapies for the purpose of remission-maintenance and advise the treatment with intravenous immunoglobulins (IVIG) in patients who have hypogammaglobulinemia (e.g., IgG < 3 g/L) and recurrent severe infections after receiving RTX^15^ while KDIGO states that a longer-term use of prophylaxis dose TMP-SMX for patients i) with structural lung disease, ii) receiving repeated rituximab infusions or iii) with ongoing immunosuppressive or glucocorticoid therapy may be considered(20). The EULAR recommendations propose measurement of IgG levels ahead of each rituximab administration, Ig replacement therapy in cases with hypogammaglobulinemia and recurrent severe infections, and the administration of prophylaxis dose TMP/SMX when RTX or CYC are used, and in the case of high-dose steroid therapy (> 15 mg/day). For patients with additional risk factors (e.g. pulmonary disease or hypogammaglobulinemia), continuation of TMP/SMX should be considered until lower doses” are achieved.

In relapsing GPA/MPA, the ACR/VF guidelines advise the switching to other remission-induction regimen, for instance, CYC if the patient received RTX for remission-induction and vice versa, but conditionally recommends remission re-induction with RTX over CYC for those patients who are not receiving RTX for remission maintenance In refractory disease, switching is also favored over combined immunosuppression and adding IVIG is also considered reasonable. In both, the EULAR and KDIGO recommendations, RTX is the preferred induction therapy in patients with a relapsing disease course. This is based on a sub-analysis of the RAVE trial (21). Treatment refractoriness should lead to reassessment of disease status and comorbidities, and consultation with centers of expertise. These patients may be managed by the addition of immunosuppressants, such as CYC after failure to achieve remission after RTX induction.
